# A Comparison of Pollination Efficiency Between Wild Bumble Bees and Introduced Honey Bees on *Polygonatum cyrtonema*

**DOI:** 10.3390/biology14030276

**Published:** 2025-03-07

**Authors:** Ju Tang, Xiang-Xiang Ge, Yu-Jie Xu, Yu Zhang, Jian-Wen Shao, Xiao-Hong Li

**Affiliations:** College of Life Sciences, Anhui Normal University, Wuhu 241002, China; tangju@ahnu.edu.cn (J.T.); gexiangxiang9912@163.com (X.-X.G.); 18255390460@163.com (Y.-J.X.); zy16822@163.com (Y.Z.); shaojw@ahnu.edu.cn (J.-W.S.)

**Keywords:** pollination efficiency, *Polygonatum cyrtonema*, bumble bees, honey bees

## Abstract

To clarify the pollination contributions of introduced honey bees and native wild bees, we compared their pollination efficiency on a perennial herb, *Polygonatum cyrtonema* Hua. The flower’s traits and bees’ body traits were measured to quantify the mechanical fit between the bee species and flower. Pollen removal and deposition per visit, visit frequency, and visit duration per flower were investigated. The results show that both native bumble bees (worker bees of *Bombus trifasciatus* Smith) and introduced honey bees (*Apis mellifera* L.) are effective pollinators, but bumble bees play a more important role in pollination, due to their higher visit frequency and slightly higher pollen transfer efficiency. The bumble bees removed and deposited significantly more pollen grains per visit than the honey bees (both *p* < 0.001). The faster visiting speed and shorter visit duration of the bumble bees, combined with their larger body size and longer proboscises, may have contributed to their higher pollen transfer efficiency. The pollination success of *P. cyrtonema* depends on its pollinators. This study is the first to report on the pollination efficiency of floral visitors on *P. cyrtonema*. Our findings highlight the importance of conserving native bumble bee populations to ensure the reproductive success of *P. cyrtonema*. Future studies should focus on their management to minimize potential disruptions to native pollination contribution.

## Simple Summary

Understanding the pollination efficiency of different pollinators is crucial for the conservation and sustainable utilization of an important medicinal resource plant, *Polygonatum cyrtonema* Hua. *P. cyrtonema* is self-compatible, but depends on pollinators. This study aimed to clarify the pollination contributions of introduced honey bees and native bumble bees. Pollination observations were conducted during peak blossom, and pollination efficiency was evaluated by determining the amount of pollen removed and deposited per visit. Generalized linear models (GLMs) were created to compare the pollination efficiency (pollen removal and deposition), visit frequency, and visit duration per flower between bumble bees (*Bombus trifasciatus* Smith) and honey bees (*Apis mellifera* L.). The results show that both native bumble bees and introduced honey bees are effective pollinators, but bumble bees are more efficient, with significantly higher pollen removal, pollen deposition, and visit frequency (all *p* < 0.001). These findings highlight the importance of native bumble bees for the reproductive success of *P. cyrtonema*, which is valuable for biodiversity conservation and plant resource management.

## 1. Introduction

Honey bees are becoming an increasingly dominant floral visitor of many crops worldwide [1,2,3]. Although native pollinator insects are usually more effective than introduced bees in some flowering plants [4,5], pollination by native bees may be influenced by introduced bees, due to factors such as pollen loss, heterospecific pollen deposition, plant reproduction, and pathogen infection [3,6]. Even the replacement of taxonomically related and functionally equivalent pollinators, such as the replacement of endemic subspecies of bumble bees by the introduction of *Bombus terrestris* L. in Japan, may reduce the fruit set of native plant species [7]. Modifications to the structure of pollination networks resulting from visitation of native plants by alien flower visitors could impose accentuated interaction costs on many native plants [6,7,8,9]. Thus, the consequences of introduced species on native pollinator systems need to be understood.

*Polygonatum* (Asparagaceae) is widely distributed in East Asia, known for its medicinal and ecological value [10,11,12]. Despite the medicinal value of plants of the genus *Polygonatum*, studies on the pollination biology of this genus are relatively limited [13,14,15], with more studies focusing on chemical composition and pharmacological analysis [16,17]. *Polygonatum cyrtonema* Hua is a perennial herbaceous flowering plant that relies on bees for successful pollination [13,14]. Although *P. cyrtonema* is capable of asexual reproduction using rhizomes, its sexual reproduction process is indispensable for promoting species adaptation to the constantly changing environment. Thanks to its rich pharmacological properties, it has shown enormous potential in the treatment of COVID-19 [18]. Moreover, it is used to concoct a variety of food products, including sweetmeats, functional beverages, and fruit wine [10,19]. However, the pollination characteristics of *P. cyrtonema*, especially regarding the performance of different pollinators, are still not well understood. Bumble bees and honey bees are the primary floral visitors of *P. cyrtonema* [13,14]. However, their pollination contributions remain unquantified. The effectiveness of honey bees (*Apis mellifera* L.) as pollinators for *P. cyrtonema* has been questioned in comparison with that of native bumble bees. Studies on tomatoes and blueberries have demonstrated that bumble bees are often more efficient pollinators, due to their capacity to transfer a larger number of pollen grains and significantly increase the fruit set compared with honey bees [20,21]. Considering the significant value of *P. cyrtonema* in the medical and food industries, as well as the importance of pollination for its sexual reproduction and species adaptation, a comprehensive understanding of its pollination process is essential. Such knowledge is crucial for optimizing pollination management strategies and ensuring the sustainable utilization of this species.

To investigate the insect visitors of *P. cyrtonema* and analyze the performance of introduced honey bees and native bumble bees, pollen removal and stigmatic pollen deposition per visit were evaluated to assess their pollination efficiency. In addition, we recorded the flower visit duration and visit frequency. Bumble bees usually have long proboscises, and their body sizes are generally bigger than those of honey bees. To examine the mechanical matching between bee visitors and flowers, the bees’ body traits (such as body length, proboscis length, head length/width, mesosoma length/width, and metasoma length/width) and the flower’s traits (floral length, corolla opening, floral diameter, stamen length, pistil length, and stigma–anther distance) were quantified as well. The aim of this study was to compare the pollination efficiency of native bumble bees (*Bombus trifasciatus* Smith) and introduced honey bees (*Apis mellifera*) on *P. cyrtonema*. We address the following three questions: (1) What insect species are effective pollinators for *P. cyrtonema*? (2) Are native bumble bees more efficient than introduced honey bees at pollinating *P. cyrtonema*? (3) How do the differences in foraging behavior and body morphologies between bumble bees and honey bees influence their pollination efficiency?

## 2. Materials and Methods

### 2.1. Study Species and Site

**Figure 1:**
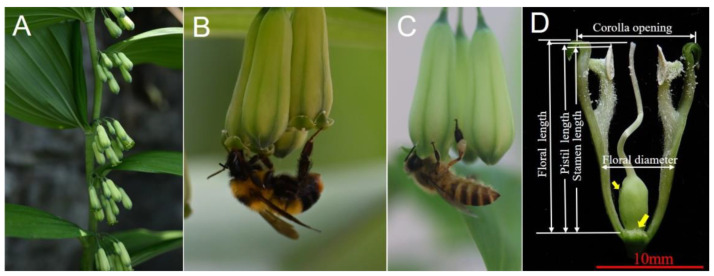
(**A**) The inflorescences of *P. cyrtonema*; (**B**) a native bumble bee *(B. trifasciatus*) and (**C**) an introduced honey bee (*A. mellifera*) foraging on the flowers of *P. cyrtonema*; and (**D**) the anatomic structure and the floral traits of a single flower. The yellow arrows show the nectar droplets.

*Polygonatum cyrtonema* Hua. (*Polygonatum* Mill., Asparagaceae) is a monoecious perennial herb [10,12] with a wide natural distribution across the forests, thickets, and shaded slopes of Anhui, Fujian, Guangdong, Guangxi, Guizhou, Henan, Hubei, Hunan, Jiangsu, Jiangxi, Shaanxi, Sichuan, and Zhejiang Provinces, China [10,22]. Anhui Province is one of the main production areas, within which Qingyang County is a geo-authentic production area [12]. The rhizome of *P. cyrtonema* is usually moniliform or tuberous moniliform, and it has an erect glabrous stem with a height of 50–100 cm [22]. The inflorescences are umbel-like, and the flowers are campanulate–cylindric with nectar (Figure 1A,D). The nectary is located at the base of the corolla (see the yellow arrows in Figure 1D). Each tubular flower consists of six anthers and one pistil (Figure 1A,D). Each flower produces more than ten ovules (13.9 ± 0.5, unpublished data), and the floral perianth is yellowish-green (Figure 1). *P. cyrtonema* relies on floral visitors to transfer pollen between its flowers for reproduction [13,14]. The lifespan of a single flower usually lasts about 5–6 days (per. obs.). All anthers fully dehisce within 48 h after flower opening [13], ensuring pollen availability during the peak period of pollinator activity. The flowers exhibit homogamy, with overlapping male (pollen viability) and female (stigma receptivity) phases [13,15]. The population flowering period is from April to June every year, while the peak blooming period is in May (unpublished data).

*Apis mellifera* Linnaeus (Apidae) is a non-native species introduced outside its native range for commercial pollination and honey production [3,23]. *A. mellifera* is a generalist pollinator. Its foraging efficiency on narrow-tubed species is often lower than that of native bumble bees [24]. *Bombus trifasciatus* Smith (Apidae) is a native bumble bee species that is widely distributed in China [25]. It is a pollinator of alpine and subalpine ecosystems, particularly for plants with tubular flowers, such as *P. cyrtonema*, due to its long proboscis. Field observations and experimental manipulations were conducted in May of 2023 and 2024 in the Baima Botanical Garden, Qingyang County, Chizhou City, Anhui Province, China (30°63′N, 117°84′E, 440 m above sea level). The specific location was on a hillside with approximately a thousand *P. cyrtonema* individuals (cultivation base of *P. cyrtonema*). The managed *A. mellifera* colonies are active throughout the flowering season, overlapping with the flowering period of *P. cyrtonema*. The cultivation base is surrounded by several other flowering species and forest species, such as *Liriope spicata* Lour. (Asparagaceae), *Salvia japonica* Thunb. (Lamiaceae), *Houttuynia cordata* Thunb. (Saururaceae), *Euonymus alatus* (Thunb.) Siebold (Celastraceae), and *Pinus* L. (Pinaceae) species.

### 2.2. Measurement of Flower’s Traits and Bees’ Body Traits

A mechanical fit between the flower and pollinators could improve the pollination efficiency in relation to realized precision and accuracy. To examine the traits matching the flowers and visitors, opening flowers were randomly sampled from twenty inflorescences (three flowers per inflorescence and sixty flowers in total). Six flower traits—floral length, corolla opening, floral diameter, stamen length, pistil length, and stigma–anther distance—were measured (Figure 1C). These traits can be used to infer the flower size and the spacing between the stamens and pistils.

**Figure 2:**
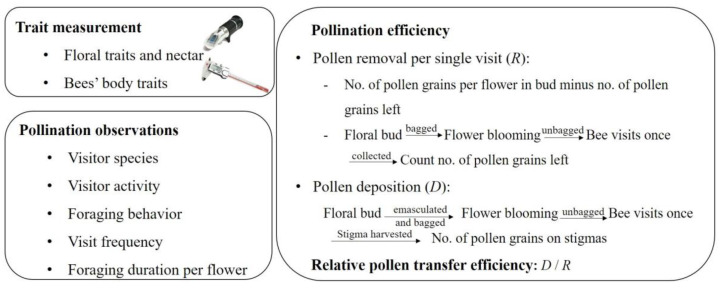
A schematic diagram of the pollination experiments with bumble bees and honey bees.

Measurements of the flower’s traits and nectar characteristics are crucial for understanding the pollination process and the foraging behavior of pollinators of *P. cyrtonema*. To investigate the nectar volume and sugar concentration of *P. cyrtonema*, 60 flowers from 20 different inflorescences were randomly labeled (3 flowers per inflorescence) and bagged during the first 24 h of anthesis. Nectar was collected 24 h later using glass microcapillary tubes. We collected nectar by inserting microcapillary tubes into the base of the flowers, avoiding damaging the tissue and contaminating the nectar sample. The nectar was naturally drawn into the tubes due to capillary pressure. To calculate the nectar volume (μL), we used the formula V_nectar_ = L/L_total_ ∗ V_total_, where L_total_ is 10 cm and V_total_ is 7 μL. The length (L) of the microcapillary tube occupied by nectar was measured with a caliper micrometer. Nectar sugar concentration (%) was measured with a handheld refractometer (Eclipse 45–81, measuring range 0–50%, Bellingham & Stanley, UK, Figure 2).

All bee specimens (*B. trifasciatus* and *A. mellifera*) were collected during the peak flowering period of *P. cyrtonema* (mid-May in 2024) at a specific time of day (09:00–17:00). Both bumble bees and honey bees were captured with 50 mL centrifuge tubes containing ethyl acetate when they visited *P. cyrtonema* flowers. To compare the differences in the body traits of bumble bees and honey bees, we measured the bees’ body traits (12 bumble bees and 10 honey bees) with an electronic digital vernier caliper (0.01 mm, Guilin Guanglu Measuring Instrument Co., Ltd., Guilin, China, Figure 2), including their body length, head length and width, proboscis length (only glossa length), mesosoma length and width, and metasoma length and width. Specimens were collected after all field pollination observations and pollination efficiency experiments had been conducted, to avoid disrupting natural pollinator behavior.

### 2.3. Pollination Observation Experiments

To investigate the visitor species and foraging behavior (for pollen or/and nectar) of *P. cyrtonema*, we conducted pollinator observations in the area where *P. cyrtonema* was seen on sunny days in the peak blooming period (on 9–11 May 2024 and 6–11 May 2023), between 8:00 and 11:30 in the forenoon and between 12:30 and 17:30 in the afternoon. A schematic diagram of the pollination observation experiments is shown in Figure 2. Two observers set up two flower plots (1 m × 1 m), with each plot containing more than five inflorescences (>100 flowers), to observe the species identity and foraging behavior of each bee species. We recorded the types of visitors and whether the insects came into contact with the anthers and stigmas. Bee visitations to a plot were recorded in 30 min sessions. A total of 67 observation sessions (33.5 h) were conducted. To evaluate which floral visitors were effective in pollination, we recorded the number of visits and the foraging behaviors of each flower visitor. After each census, the number of open flowers in each observation plot was counted, and the visit frequency was calculated as the number of visits per flower per hour. To determine the foraging behavior of the bees, we observed which reward they were foraging for, and recorded whether the bees were collecting pollen grains. We also recorded where pollen landed on the body during foraging.

### 2.4. Pollination Efficiency of Bumble Bees and Honey Bees

To compare the pollination efficiency between bumble bees and honey bees, we investigated pollen removal and deposition during a single visit (Figure 2). For the assessment of pollen removal, at least 100 buds from 20 inflorescences of *P. cyrtonema* were bagged in fine-mesh gauze bags. Once the flowers had fully bloomed, the bags were removed and the flowers were exposed to potential pollinators between 9:00 and 17:00. Then, the observers carefully monitored the flowers to ensure that each flower was visited only once by either a bumble bee or a honey bee. Each flower was stored in a 2 mL centrifuge tube with 75% alcohol. In total, we collected 27 flowers visited by bumble bees and 24 flowers visited by honey bees. We collected 40 floral buds with undehisced anthers to count the pollen production per flower. We measured the pollen counts (83,695.0 ± 2264.1, mean ± SE, unpublished data). The CV of pollen production per flower was 0.1711. The number of pollen grains remaining in the flowers were counted under a microscope. Pollen removal per flower was calculated as the number of pollen grains per flower in undehisced anthers, minus the number of pollen grains remaining in the once-visited flower (Figure 2).

To quantify pollen deposition on stigmas during a single visit, more than 100 flowers buds were emasculated by a pair of forceps before the anthers were dehisced and bagged. When these flowers entered their female phase, we removed the bags and waited for the bees to visit. Their stigmas were harvested once a bumble bee or honey bee had visited. The stigmas were then stored in 0.2 mL centrifuge tubes filled with 75% alcohol. In total, we harvested 34 stigmas visited by bumble bees and 45 stigmas visited by honey bees. All the stigmas were softened in 8 Mol/L NaOH solutions for two hours, and the pollen grains on the stigmas were counted under a light microscope after being dyed with a safranin stain solution in a laboratory. The pollen grains deposited during a single visit, including those that dropped off the stigmas into the tube during storage or into the NaOH solution, were considered. We used pollen deposition divided by pollen removal to quantify the relative pollen transfer efficiency (D/R) of bumble bees and honey bees [26,27].

### 2.5. Data Analysis

Generalized linear models (GLMs) were used to analyze all data, including body traits, pollen removal, pollen deposition, visit frequency, and visit duration per visit, due to their flexibility in handling non-normal distributions and different data types. To compare the body traits of bumble bees and honey bees, we used a GLM with a normal distribution and an identity-link function. The body traits were the dependent variables, and the species was the independent variable. To compare the visitation frequency of bumble bees and honey bees, we used a GLM with a normal distribution and an identity-link function. The visitation frequency was the dependent variable, and the bee species was the independent variable. To compare the visit duration per flower between the bumble bees and honey bees, visit duration (as the dependent variable) and bee species (as the independent variable) were analyzed using a GLM with a normal distribution and an identity-link function. The number of pollen grains removed and deposited per visit were considered count data, which often exhibit over-dispersion. To compare pollination efficiency, the data were analyzed with a GLM with a Poisson distribution and a loglinear-link function. The number of pollen grains removed and deposited per visit were the dependent variables, and the bee species was the independent variable. All data analyses were performed in SPSS 22.0 (IBM, New York, NY, USA). OriginPro v.9.5 and Photoshop CS6 13.0 were used for graph plotting.

## 3. Results

### 3.1. Flower’s Traits and Bees’ Body Traits

The flowers of *P. cyrtonema* are tubular, and the inflorescences are umbel-like (Figure 1A). The longevity of a single flower is 5–6 days. The floral length is 20.06 ± 0.18 mm (N = 60), and the corolla opening is 8.66 ± 0.08 mm (Table 1). Each flower has six stamens, which are situated around the style (Figure 1D). Although the pistil curves upward, the pistil length is longer than the anther length and the flower is herkogamous (Table 1; Figure 1A,D). The nectary is located at the base of the corolla, and nectar is secreted throughout the flowering duration. Each flower can secrete 13.96 ± 0.92 μL of nectar in a 24 h period during the first flowering day, with approximately 32.79 ± 0.75 g/100 mL of sugar concentration for bee foragers (Table 1).

When comparing the body traits of bumble bees and honey bees, we found that the body traits of bumble bees are significantly larger than those of honey bees (Table 2, all *p* < 0.001). The head widths of both bumble bees (4.48 ± 0.14, N = 12) and honey bees (3.79 ± 0.03, N = 10) are smaller than the floral opening (8.66 ± 0.08 mm) and diameter (5.46 ± 0.05, N = 60), suggesting that both bees can forage in the corolla (Figure 1B,C). The proboscis length of honey bees (5.43 ± 0.21 mm, N = 10) is significantly shorter than that of bumble bees (9.38 ± 0.37 mm, N = 12), and the floral length (20.06 ± 0.18 mm, N = 60) is longer than the proboscis length of both bees (Table 2). These size relationships indicate that both bees can access the interior of the flower corolla tube, which is beneficial for their activities as they can forage for nectar and contact the anthers and stigmas.

### 3.2. Pollination Observations

Our field pollinator observations (a total of 34 sessions, totaling 17 h, in 2023, and 33 sessions, totaling 16.5 h, in 2024) indicated that bumble bees (worker bees of *Bombus trifasciatus* Smith) and honey bees (*Apis mellifera* L.) are the predominant floral pollinators of *P. cyrtonema*.

**Figure 3:**
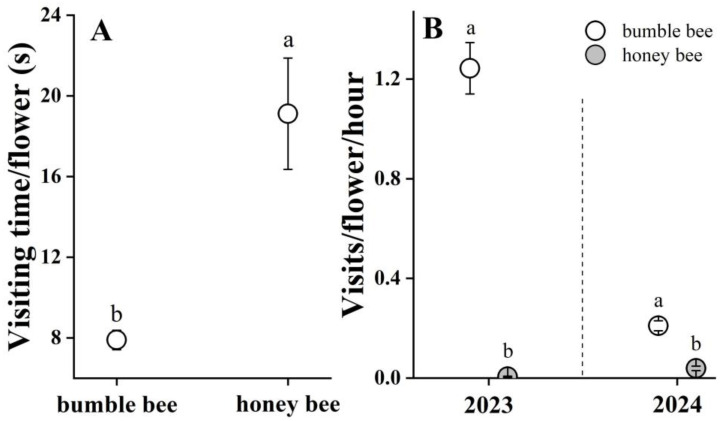
(**A**) Duration of visits to *P. cyrtonema* per flower (in seconds). Sample sizes for each bee are as follows: N = 35 bumble bees and N = 25 honey bees. (**B**) Frequency of visits by bumble bees (open circles) and honey bees (gray circles) in 2023 and 2024. The different letters (a and b) indicate statistically significant differences at *p* < 0.05 with the GLMs analysis.

Both bumble bees (*B. trifasciatus*) and honey bees (*A. mellifera*) were observed to enter the floral tube of *P. cyrtonema* to forage nectar. Bumble bees inserted their proboscises into the tube while clinging to the tepals with their legs, whereas honey bees exhibited shorter proboscis extensions, and often foraged from the tube entrance (Figure 1B,C). Bumble bees foraged for nectar with their long proboscises, and occasionally autogroomed by removing the pollen grains from the head and mesosoma, while honey bees collected pollen grains and packed the pollen loads onto their hind legs (Figure 1C). The visit duration per flower for bumble bees (7.90 ± 0.48 s, N = 35) was significantly shorter (Wald χ^2^ = 22.010, *p* < 0.001) than that for honey bees (19.12 ± 2.76 s, N = 25, Figure 3A). The combined two-year observations showed that the mean visit frequency (visits/flower/hour) was significantly higher (*p* < 0.001) for bumble bees (1.25 ± 0.11, N = 34 in 2023 and 0.21 ± 0.02, N = 33 in 2024) than for honey bees (0.01 ± 0, N = 34, in 2023, Wald χ^2^ = 54.032, *p* < 0.001; 0.04 ± 0.01, N = 33 in 2024, Wald χ^2^ = 54.032, *p* < 0.001). In addition, we found that bumble bees are active throughout the day, whereas honey bees are mainly active between 10:00 and 15:00 (Figure S1).

### 3.3. Pollination Efficiency

**Figure 4:**
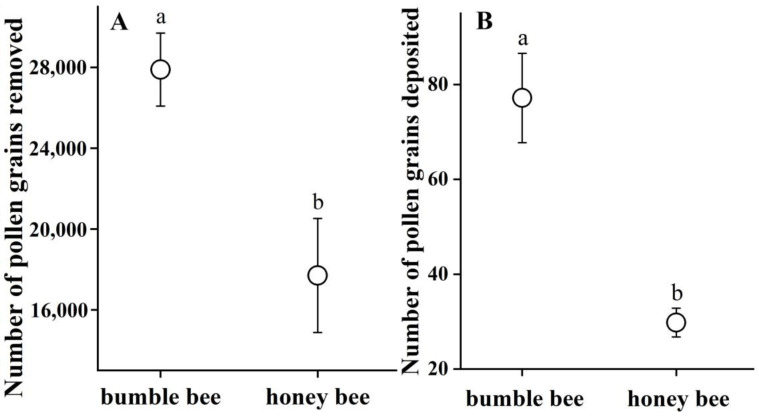
(**A**) Pollen removal and (**B**) pollen deposition (mean ± S.E.) during single visit to *P. cyrtonema* by bumble bees and honey bees. Sample sizes of flowers (N) are as follows: N = 27 bumble bees and N = 24 honey bees for pollen removal, and N = 34 bumble bees and N = 45 honey bees for pollen deposition. The different letters (a and b) indicate statistically significant differences at p < 0.05 with the GLMs analysis.

All pollen grains were released onto the head and ventral part of the bees’ bodies around the areas where the stigmas were attached (Figure 1B,C). Bumble bees removed significantly more pollen grains per visit (27,891.3 ± 1806.0, N = 27) than honey bees (17,697.1 ± 2821.1, N = 24) (Wald χ^2^ = 8.242, df = 1, *p* = 0.004; Figure 4A), and deposited significantly more pollen grains (77.2 ± 9.4, N = 34) than honey bees (29.8 ± 3.0, N = 45) (Wald χ^2^ = 36.933, *p* < 0.001) (Figure 4B), indicating that the pollination efficiency of bumble bees is significantly higher than that of honey bees. The relative pollen transfer efficiency (D/R) of bumble bees (0.0028) was higher than that of honey bees (0.0017).

## 4. Discussion

Our investigations showed that *P. cyrtonema* was pollinated by both bumble bee and honey bee workers. The measurements of pollination effectiveness and efficiency showed that the bumble bees were both more effective (deposited more pollen per visit, 77.2 ± 9.4 vs. 29.8 ± 3.0, *p* < 0.001) and more efficient (deposited a higher proportion of the pollen removed, 0.0028 vs. 0.0017). Therefore, bumble bees play important roles in the pollination of *P. cyrtonema*, due to their higher visit frequency (Figure 3B) and pollination efficiency (Figure 4).

Flowering plants are usually visited by diverse insects; however, not all of these visitors are effective pollinators [28]. Different floral visitors vary significantly regarding the extent to which they contribute to plant pollination [5,29,30]. Differentiating pollinators from other floral visitors is thus crucial for understanding plant reproductive success [28]. Quantifying the pollination contribution of native and introduced pollinators helps to optimize the management of introduced insect species [3,31]. In our study, bumble bees and honey bees were identified as the primary pollinators of *P. cyrtonema* (Figure 1B,C). Both bees visited the flowers and could transfer their pollen grains successfully (Figure 1B,C, Figure 4 and Figure S1). However, other visitors such as *B. flavescens* Smith and syrphid flies only visited occasionally (one time for *B. flavescens* and seven times for syrphid flies during our observations). These occasional visitors made ineffective visits, and they landed on the leaves or corollas of *P. cyrtonema*. Syrphid flies stayed for a long time, but rarely touched the anthers and stigmas of *P. cyrtonema* simultaneously (per. obs.). Native bumble bees were the most effective pollinators for *P. cyrtonema*, thanks to their higher visit frequency (0.74 ± 0.08, mean ± S.E. for 2023 and 2024) and foraging activity throughout the day (Figure S1).

As an important medicinal and dietary economic plant, *P. cyrtonema* has no autogamy and apomixis systems, highlighting its reliance on pollinators for sexual reproduction [13,14,15]. Under natural conditions, *P. cyrtonema* suffers severe flower and fruit drop (pers. obs.), which may be related to factors such as environmental stress, resource allocation constraints, and pollination service effectiveness [32]. This further emphasizes the significance of efficient pollination for its fruit production. Previous studies considered bumble bees and honey bees as pollinators of *P. cyrtonema* [13,14], but our study is the first to compare their pollination efficiency in terms of pollen removal, deposition per visit, and visit frequency. Honey bees (*A. mellifera*) are important pollinators in orchards and crops [1,2,3,33]. However, their pollination efficiency is often lower than that of other native bees for almonds [34], apples [35], and cherries [36], although their numerical dominance sometimes enhances their pollination contribution [24,37]. However, in our study, bumble bees showed a significantly higher visit frequency (Figure 3B) and pollination efficiency (Figure 4) than honey bees. Consistently with reports that native pollinators are often more effective than introduced ones [4,5], we found that native bumble bees (worker bees of *B. trifasciatus*) play a leading role in pollen transfer for *P. cyrtonema* flowers. However, as generalist foragers, honey bees visit multiple co-flowering species. Even though they are less effective pollinators for *P. cyrtonema*, their introduction may still benefit other flowering plants [3]. Furthermore, with increased pollinator density, which might be a strong selective agent acting on wild plant populations, introduced honey bees could affect the evolution of species interactions and plant reproductive traits, such as nectar volumes and floral traits [38,39]. The competition between native bumble bees and introduced honey bees for floral resources could decrease the species richness and abundance of native bumble bees and negatively affect native plant–pollinator interactions [39].

The pollination contribution of bees is commonly evaluated based on pollination efficiency and visit frequency (i.e., pollination quality and pollination quantity, respectively) [29,30,31,32,40,41,42,43]. Pollination efficiency is often measured in terms of the number of pollen grains transferred by the pollinator per visit [5,41,42,43], and varies among different pollinator species and ecosystems [3,31]. In this study, we quantified pollination efficiency as the pollen deposited on stigmas and pollen removed from anthers per visit (Figure 2), while we used the ratio of pollen removed and deposited per visit to quantify relative pollen transfer efficiency, as this metric reflects the effectiveness of pollinators in transferring pollen to receptive stigmas. Due to the formation of inflorescences of many flowers on the same plant of *P. cyrtonema*, avoiding cross-pollination within the same plant is difficult. It should be noted that our measurements of pollen deposition did not distinguish between xenogamous (cross-pollination) and geitonogamous (within-plant pollination) pollen transfer. Future studies could address this limitation by using quantum dot markers [44] to differentiate pollen sources and compare the xenogamous abilities between bumble bees and honey bees.

Different pollinator insects have developed different adaptive behaviors and morphological mechanisms relating to flowers. The size and shape of a pollinator’s body can determine how much pollen it can carry and how well it can access the anthers and stigmas of a flower [45,46,47,48,49]. Pollinators with greater body sizes have been reported to carry more pollen grains and deposit more pollen grains on stigma in oilseed rape *Brassica napus* [50] and watermelon *Citrullus lanatus* [51]. In this study, the superior pollination performance of native bumble bees (*B. trifasciatus*) over introduced honey bees (*Apis mellifera*) for *P. cyrtonema* can be attributed to their morphological traits. Bumble bees have larger body sizes (16.57 ± 0.62 mm) and longer proboscises (9.38 ± 0.37 mm, Table 2), which enable them to access floral resources more effectively and enhance pollen transfer [45]. The normalized values (pollen grains per mm of body length) confirm that bumble bees are more efficient pollinators. The bumble bees deposited significantly more pollen grains per body size (4.7 ± 0.6 grains/mm) than the honey bees (2.4 ± 0.2 grains/mm, *p* < 0.001, GLM). Bumble bees removed 1683.2 ± 109.0 grains/mm, while honey bees removed 1427.2 ± 227.5 grains/mm (*p* = 0.309 > 0.05). The body size of bumble bees provides a better mechanical fit with the floral traits of *P. cyrtonema* (Figure 1B,C; Table 1 and Table 2). Their long proboscises (9.38 ± 0.37 mm) allow them to reach the nectar at the base of the corolla (20.06 ± 0.18 mm) more easily. During this process, the increased contact with the anthers and stigmas significantly increases the chances of pollen grain transfer (Figure 1 and Figure 4).

Additionally, their foraging behavior, such as faster movements between flowers and shorter visit duration (7.90 ± 0.48 s, Figure 3A), could contribute to their higher pollinator effectiveness compared with that of honey bees (Figure 4). This is inconsistent with the conclusion that, in some plants, the longer an insect spends handling a flower, the more pollen will be deposited on the insect’s body and the stigma [30,50]. The reason for this is likely related to the behavior of both bees. When the bumble bees visited the flowers, their loud buzzing sounds and the shaking of the flower upon landing or leaving were associated with active pollen collection. By vibrating their flight muscles while clinging to the flowers, the bumble bees dislodged pollen from the anthers of *P. cyrtonema*, which resulted in lots of pollen grains being released onto their bodies (per. obs.). In contrast, the honey bees did not exhibit this behavior, and instead passively collected pollen from the anther surfaces. The body size of the honey bees might also have limited their pollen transfer capabilities. Furthermore, honey bees’ frequent grooming behavior, which transfers pollen to their hind legs for storage, may reduce their pollen transfer efficiency [46], because both the plants and bees compete for the pollen [52]. In corbiculate bees, pollen is moistened with nectar and packed into the corbicula (pollen baskets) for transport back to their nest, where nearly all of it will be used to feed larvae. Once in the corbicula, the pollen is no longer available for pollination. This reduces the amount of pollen available for plant reproduction, thereby decreasing the male fitness of plants [53].

## 5. Conclusions

Our study comprehensively examined the pollination of *P. cyrtonema*, a plant of significant medicinal and economic value. We clarified that both native bumble bees (worker bees of *B. trifasciatus*) and introduced honey bees (*A. mellifera*) are pollinators for *P. cyrtonema*, but bumble bees exhibit higher pollination efficiency, due to their larger body size, longer proboscis, faster visiting speed, and shorter visit duration leading to greater pollen removal and deposition, i.e., higher pollination efficiency. Their frequent visits ensure effective pollination for *P. cyrtonema*. As *P. cyrtonema* relies on pollinators for reproduction, and introduced species may negatively impact native pollination systems through pollen loss and pathogen transmission [3], conserving native bumble bee populations is crucial for the sustainable utilization of *P. cyrtonema*. Our research demonstrates that native bumble bees are more effective pollinators of *P. cyrtonema* than introduced honey bees. This highlights the importance of conserving native pollinator populations to ensure the reproductive success of *P. cyrtonema* and other native plants. Future research should focus on the long-term monitoring of pollination dynamics, particularly exploring the broader ecological consequences of introduced honey bees on native plant–pollinator networks.

## Figures and Tables

**Table 1:** Floral traits and nectar volume and concentration of *Polygonatum cyrtonema* (N = 60).

Floral Traits	Mean ± S.E.
Floral length (mm)	20.06 ± 0.18
Corolla opening (mm)	8.66 ± 0.08
Floral diameter (mm)	5.46 ± 0.05
Stamen length (mm)	18.70 ± 0.17
Pistil length (mm)	19.69 ± 0.20
Stigma–anther distance (mm)	0.82 ± 0.03
Nectar volume (μL)	13.96 ± 0.92
Nectar concentration (%)	32.79 ± 0.75

**Table 2:** Differences in body traits between bumble bee and honey bee visitors of *Polygonatum cyrtonema*. The different letters (a and b) indicate statistically significant differences in bees’ body traits at *p* < 0.05 with the GLMs analysis.

(mm)	Head Length	Head Width	Proboscis Length	Mesosoma Length	Mesosoma Width	Metasoma Length	Metasoma Width	Body Length
Bumble bee (N = 12)	5.60 ± 0.24 a	4.48 ± 0.14 a	9.38 ± 0.37 a	6.01 ± 0.16 a	5.81 ± 0.18 a	8.09 ± 0.50 a	6.82 ± 0.26 a	16.57 ± 0.62 a
Honey bee (N = 10)	3.83 ± 0.05 b	3.79 ± 0.03 b	5.43 ± 0.21 b	3.94 ± 0.07 b	4.04 ± 0.06 b	6.14 ± 0.12 b	4.16 ± 0.07 b	12.40 ± 0.15 b
Wald χ^2^	44.758	19.809	76.391	115.645	76.47	12.188	80.491	35.506
*p*	<0.001	<0.001	<0.001	<0.001	<0.001	<0.001	<0.001	<0.001

## Data Availability

Data are contained within the article and Supplementary Materials.

## Supplementary Materials

The following supporting information can be downloaded at https://www.mdpi.com/article/10.3390/biology14030276/s1: Figure S1. Frequency of visits to *P. cyrtonema* by bumble bees (circle) and honey bees (triangle) in different 30 min observation sessions (from 8:30 to 11:30 and 12:30 to 17:30) in 2023 (open) and 2024 (closed), on days with fine weather.
