# Supplementary material for: A Comparison of Pollination Efficiency Between Wild Bumble Bees and Introduced Honey Bees on Polygonatum cyrtonema
Source: Biology (Basel). 2025 Mar 7;14(3):276. doi: 10.3390/biology14030276 (PMC11940143; doi:10.3390/biology14030276)
Supplement: Supplementary file 1 [file biology-14-00276-s001.zip › biology-3467097-supplementary.pdf]

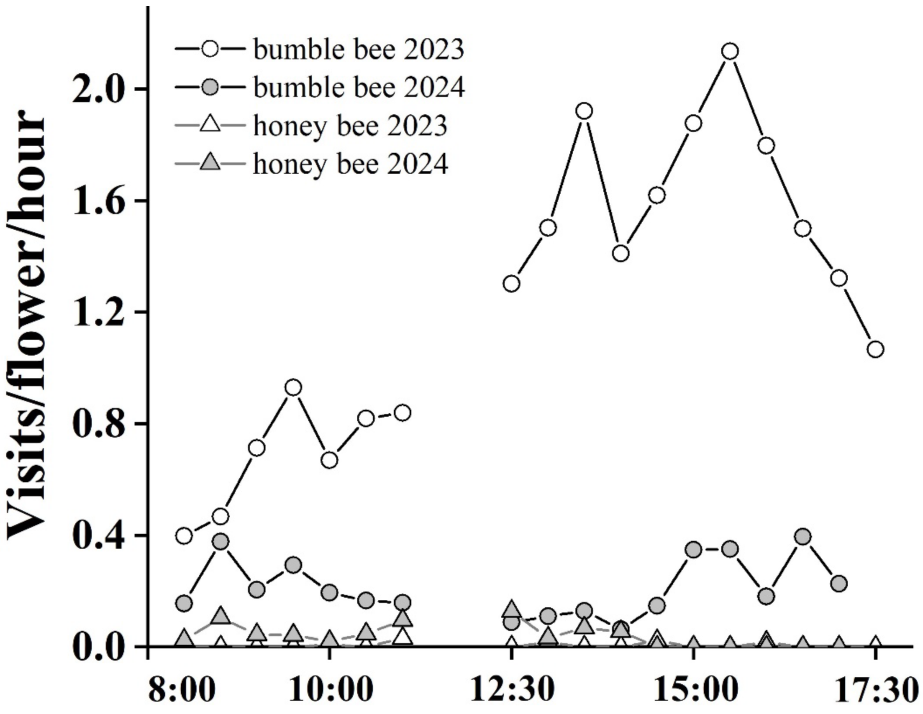

Figure S1. Frequency of visits to *P. cyrtonema* by bumble bees (circle) and honey bees (triangle) in different 30 min observation sessions (from 8:30-11:30 and 12:30-17:30) in 2023 (open) and 2024 (closed) on days with fine weather.
